# Improved Kidney Allograft Function after Early Conversion of Fast IR-Tac Metabolizers to LCP-Tac

**DOI:** 10.3390/jcm11051290

**Published:** 2022-02-26

**Authors:** Gerold Thölking, Filiz Tosun-Koç, Ulrich Jehn, Raphael Koch, Hermann Pavenstädt, Barbara Suwelack, Stefan Reuter

**Affiliations:** 1 Department of Internal Medicine and Nephrology, University Hospital of Münster Marienhospital Steinfurt, 48565 Steinfurt, Germany; filiz.tosun-koc@ukm-mhs.de; 2 Department of Medicine D, Division of General Internal Medicine, Nephrology and Rheumatology, University Hospital of Münster, 48149 Münster, Germany; ulrich.jehn@ukmuenster.de (U.J.); hermann.pavenstaedt@ukmuenster.de (H.P.); barbara.suwelack@ukmuenster.de (B.S.); stefan.reuter@ukmuenster.de (S.R.); 3 Institute of Biostatistics and Clinical Research, University of Münster, 48149 Münster, Germany; raphael.koch@ukmuenster.de

**Keywords:** kidney transplantation, renal, LCPT, tacrolimus, metabolism, C/D ratio, conversion, switch

## Abstract

Fast tacrolimus (Tac) metabolism is associated with a more rapid decline of renal function after renal transplantation (RTx). Because the pharmacokinetics of LCP-Tac (LCPT) and immediate-release Tac (IR-Tac) differ, we hypothesized that switching from IR-Tac to LCPT in kidney transplant recipients would improve the estimated glomerular filtration rate (eGFR), particularly in fast metabolizers. For proof of concept, we performed a pilot study including RTx patients who received de novo immunosuppression with IR-Tac. A Tac concentration-to-dose ratio (C/D ratio) < 1.05 ng/mL·1/mg defined fast metabolizers and ≥1.05 ng/mL·1/mg slow metabolizers one month after RTx. Patients were switched to LCPT ≥ 1 month after transplantation and followed for 3 years. Fast metabolizers (*n* = 58) were switched to LCPT earlier than slow metabolizers (*n* = 22) after RTx (2.0 (1.0–253.1) vs. 13.2 (1.2–172.8) months, *p* = 0.005). Twelve months after the conversion to LCPT, Tac doses were reduced by about 65% in both groups. The C/D ratios at 12 months had increased from 0.66 (0.24–2.10) to 1.74 (0.42–5.43) in fast and from 1.15 (0.32–3.60) to 2.75 (1.08–5.90) in slow metabolizers. Fast metabolizers showed noticeable recovery of mean eGFR already one month after the conversion (48.5 ± 17.6 vs. 41.5 ± 17.0 mL/min/1.73 m², *p* = 0.032) and at all subsequent time points, whereas the eGFR in slow metabolizers remained stable. Switching to LCPT increased Tac bioavailability, C/D ratio, and was associated with a noticeable recovery of renal function in fast metabolizers. Conversion to LCPT is safe and beneficial early after RTx.

## 1. Introduction

During the last decades, tacrolimus (Tac) has become the most commonly prescribed calcineurin inhibitor (CNI) after solid organ transplantation [1,2]. Three different Tac formulations are available. Immediate-release Tac (IR-Tac) was the first formulation on the market and has to be administered twice-daily, resulting in two blood peak levels: a higher one in the morning, about 1.5 to 2 h after drug intake, and a lower one about 12 h later [3]. Secondly, extended-release Tac (ER-Tac) was developed to simplify administration by replacing croscarmellose in IR-Tac with ethylcellulose in ER-Tac. The formulation of ER-Tac allows for once-daily administration, resulting in higher adherence rates [4], but is associated with an early high Tac peak level after ingestion that is comparable to the first peak of IR-Tac [3]. Thirdly, LCP-Tac (LCPT) is a new formulation using the MeldDose^®^ prolonged-release technology that provides absorption throughout the entire gastrointestinal tract. This results in lower and later peak concentrations approximately 4 to 6 h after drug intake [3].

Several studies have reported that the treatment of fast metabolizers after RTx with IR- or ER-Tac is associated with adverse outcomes [5,6,7,8,9,10]. In 2014, we established the Tac concentration-to-dose ratio (C/D ratio) as a surrogate marker for the Tac metabolism rate [5]. Interestingly, a low C/D ratio is associated with higher peak-level concentrations and with a more rapid decline of kidney function compared to slow metabolizers (C/D ratio ≥1.05 ng/mL·1/mg) as early as one month after RTx [5,11]. In two consecutive studies, we were able to confirm the decreased renal function of fast Tac metabolizers compared with slow metabolizers in separate IR- and ER-Tac cohorts in a 5-year follow-up [6,12]. The reasons for the impairment of renal function in the group of fast metabolizers have been reported as increased rates of acute rejections (AR) [6,10], BK-virus infections and nephropathy [5,13], CNI-nephrotoxicity (CNIT) [5,12], and higher incidences of interstitial fibrosis and tubular atrophy (IF/TA) [9]. Fast Tac metabolizers were also converted earlier and more frequently to other immunosuppressive drugs compared with slow metabolizers because of Tac-associated side effects [5,12]. For example, we found a strong association between the Tac metabolism rate and the degree of biopsy-proven CNIT [11]. Recently, two studies in French and German cohorts showed increased mortality rates even in fast Tac metabolizers [6,8]. In 2020, von Einsiedel et al. published a study on liver transplant recipients (LTR) and showed an improvement in renal function after the conversion from IR- and ER-Tac to LCPT [14]. Though in this study, the Tac dose was reduced by about 30%, the C/D ratio or bioavailability increased by about 50% twelve months after the conversion.

As bioavailability, and Tac peak levels in the blood especially, differ significantly between LCPT and IR-Tac treated patients, we hypothesize that the conversion of fast IR-Tac metabolizers to LCPT results in an improvement in kidney function.

## 2. Patients and Methods

### 2.1. Study Cohort

We retrospectively analyzed RTx recipients who underwent transplantation at the University Hospital of Münster between 2005 and 2019. All patients started immunosuppression with IR-Tac and remained on the medication for at least one month after RTx. Included patients were switched to LCPT one month after RTx or later and then followed up for three years. Target Tac trough level was 6–10 ng/mL for the first month. LCPT was introduced with a 30% reduction of preconversion daily IR-Tac [3]. Co-immunosuppression consisted of mycophenolate mofefil or mycophenolate sodium and prednisolone. Induction therapy was basiliximab or thymoglobulin, depending on immunological risk.

The C/D ratio was calculated as described before [13]. A C/D ratio < 1.05 ng/mL·1/mg defined fast metabolizers, and a value ≥ 1.05 ng/mL·1/mg defined slow metabolizers. IR-Tac doses, trough levels, and C/D ratios were given at month 1 (M1) before the switch, at the day of the switch (before switch), for LCPT at day 10 (D10), and month 1-36 (M1-36) after conversion. The estimated glomerular filtration rate (eGFR) was calculated using the CKD-EPI formula [15] at D10 and M1 after RTx, at the day of the switch (before switch), and D10, M1-36 after conversion.

General demographic data on recipients and information on transplantation were obtained from electronic health records, and data on donors were taken from Eurotransplant. The study was performed in accordance with the Declaration of Helsinki and approved by the local ethics committee (Ethik Kommission der Ärztekammer Westfalen-Lippe und der Medizinischen Fakultät der Westfälischen Wilhelms-Universität, No. 2014-381-f-N). Written informed consent with regard to recording their clinical data was given by all participants at the time of transplantation or inclusion into the study. Data were anonymized before analysis. RTx recipients younger than 18 years of age and pregnant women were excluded.

### 2.2. Statistics

Statistical analyses were performed using IBM SPSS^®^ Statistics 27 for Windows (IBM Corporation, Somers, NY, USA) and SAS software, Version 9.4 TS1M7 of the SAS System for Windows (Copyright © 2021 SAS Institute Inc., Cary, NC, USA). All *p*-values and confidence limits were two-sided and were intended to be exploratory, not confirmatory. Therefore, no adjustment for multiplicity was made. Exploratory *p*-values ≤ 0.05 were considered to be statistically noticeable.

In descriptive analysis, normally distributed continuous variables are reported as mean ± standard deviation and non-normally distributed continuous variables as median (25% quantile–75% quantile, IQR). Absolute and relative frequencies are given for categorical variables. Metabolism groups were compared using Welch’s *t*-tests for normally distributed data, Mann–Whitney U tests for skewed-distributed data, and Fisher’s exact or chi-squared tests for categorical variables. A comparison of the eGFR changes within each metabolism group was performed using Wilcoxon’s signed-rank tests. Boxplots were used for graphical representation.

In order to model renal function (eGFR) over time adjusted for dropouts, a multivariable linear mixed model was fitted. The main effects of the factors, which were time since switch (Day 10, month 1, 3, 6, 9, 12, 24, and 36 after switch), metabolizer group (fast/slow), and the interaction between time and group were included as influencing variables. Repeated measurements of each patient were modeled using SAS PROC MIXED by fitting a marginal linear mixed model with an unstructured variance–covariance matrix for the residuals with patient as subject and the order given by time. The empirical sandwich covariance estimator was applied. Missing values were treated as missing at random. Results are reported as least square estimates with the corresponding 95% confidence interval (CI) and *p*-values from the Wald test.

## 3. Results

### 3.1. Study Cohort

**Figure 1:**
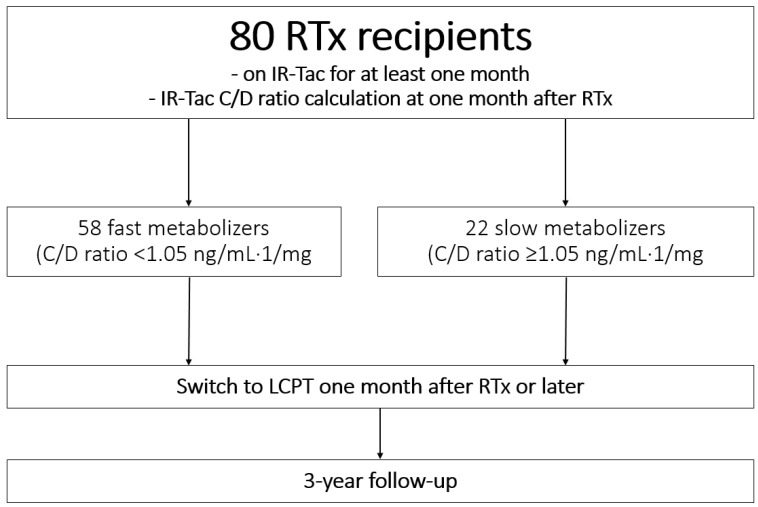
Study design and patient enrolment. A total of 80 renal transplant recipients met the inclusion criteria. RTx recipients were defined as fast and slow Tac metabolizers one month after transplantation. All patients were switched to LCPT and observed in a 3-year follow-up. Abbreviations: RTx, renal transplantation; IR-Tac, immediate-release tacrolimus; C/D ratio, concentration-to-dose ratio.

In total, we followed up 80 RTx recipients who started on IR-Tac and were switched to LCPT one month after RTx or later. A total of 58 patients were characterized as fast and 22 as slow metabolizers according to their corresponding Tac C/D ratio at one month after RTx (Figure 1). Baseline characteristics at RTx did not differ noticeably between the groups (Table 1). There were no noticeable differences between the groups in terms of underlying diseases leading to end-stage renal disease. Most of the patients had arterial hypertension at the time of RTx. In average, fast Tac metabolizers were converted earlier to LCPT compared to slow metabolizers (0.2 (1.0–253.1) vs. 13.2 (1.2–172.8) months after RTx; *p* = 0.005). The main reasons for a switch in both groups were large trough level variations and avoidance of adverse effects (Table 2).

### 3.2. Immunosuppression

The immunosuppression is shown in Table 3. Prednisolone and mycophenolate doses did not differ between the metabolizer groups one month after RTx.

IR-Tac and LCPT doses of fast metabolizers were higher at all time points compared to slow metabolizers (all *p* < 0.016). After the recommended dose reduction of 30% at the day of conversion in both groups, the dose reduction in fast metabolizers was 31.7% on D10, 64.6% on M12, and 70.7% on M36. In slow metabolizers, the median Tac dose was reduced by 48.2% at D10, by 66.7% at M12, and by 70.4% at M36.

IR-Tac trough levels at month 1 and LCPT trough levels at time points D10, M1 and M6 were lower in fast metabolizers. Tac trough levels of fast metabolizers increased slightly after the conversion and achieved the level of the trough concentration “before conversion” approximately at the 12 months mark after conversion (M12). In the group of slow metabolizers, trough concentrations were reduced considerably during the 36-month follow-up.

The C/D ratios of fast metabolizers were lower compared to the C/D ratios of slow metabolizers at all time points. After conversion to LCPT, the median C/D ratio in fast metabolizers increased by 38.9% at D10, 163.6% at M12 and 180.3% after M36. The C/D ratios of slow metabolizers rose by 66.1% at D10 days, 139.1% at M12 and 130.4% after 36 months.

### 3.3. Renal Function

**Figure 2:**
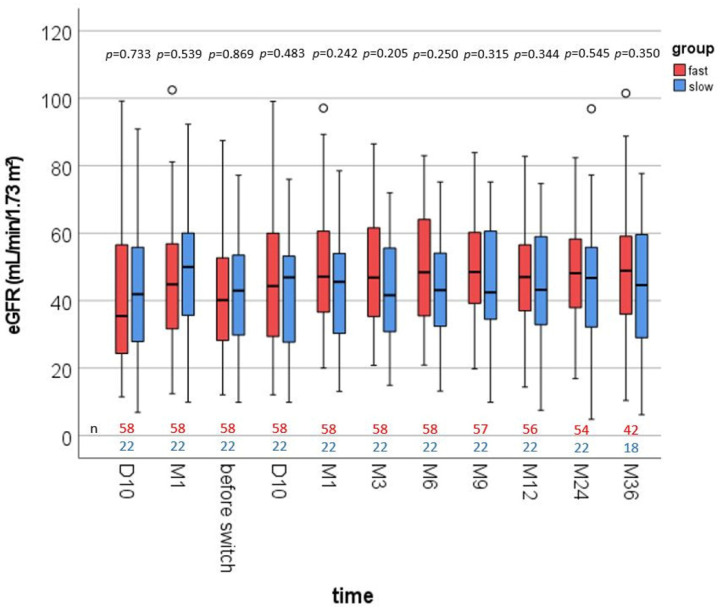
Boxplots of the renal function at different time points (eGFR, estimated glomerular filtration rate) within 3 years after kidney transplantation. *p*-values are from Mann–Whitney U tests comparing fast vs. slow metabolizers at each time point. There were no noticeable differences between the groups after conversion.

Compared with slow metabolizers, fast IR-Tac metabolizers showed a slightly but not noticeably reduced renal function one month after RTx (47.6 ± 20.8 vs. 44.4 ± 19.0 mL/min/1.73 m², respectively, *p* = 0.539, Figure 2). After conversion to LCPT, we observed similar eGFR values in both groups. In fast metabolizers, the mean eGFR increased from 41.5 ± 17.0 (before switch) to 48.5 ± 17.6 at M1, to 47.8 ± 15.4 at M12, and to 48.9 ± 19.0 mL/min/1.73 m² at M36 after the conversion. The mean eGFR values of slow metabolizers were 42.2 ± 17.5 before the switch and 43.2 ± 17.7 at M1, 43.7 ± 17.9 at M12, and 43.8 ± 19.2 mL/min/1.73 m² at M36 after the conversion.

**Figure 3:**
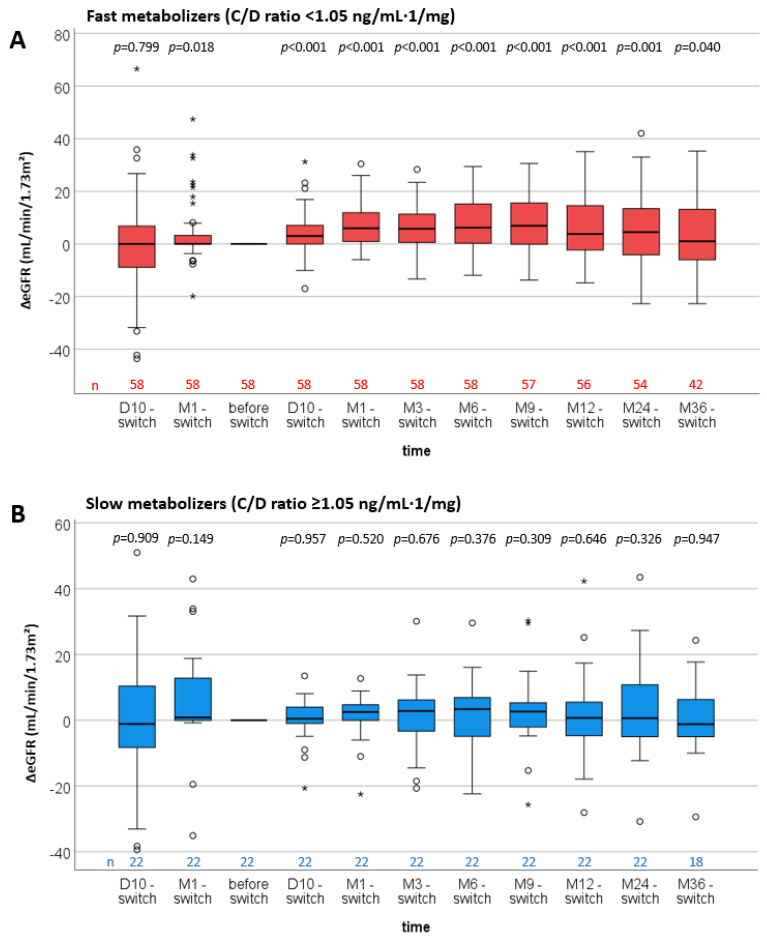
Boxplots of the differences in estimated glomerular filtration rates (ΔeGFR) of fast and slow Tac metabolizers (all time points—date of the switch). *p*-values are from Wilcoxon-signed rank tests for the dependent comparisons of eGFR values at each time point with the eGRF values at the time of the switch. The C/D ratio cut-off to characterize the metabolizer group was 1.05 ng/mL·1/mg. Fast metabolizers developed a noticeable increase in ΔeGFR at D10 after conversion and all following time points (**A**). ΔeGFR values of slow metabolizers remained stable during the 3 years after the switch (**B**).

In addition, the differences in eGFR between the “before switch” time point and all other time points (ΔeGFR) were analyzed (Figure 3). In fast metabolizers, the ΔeGFR between “M1 before switch” and the date “before switch” differed noticeably (Figure 3A). Additionally, all ΔeGFR values following the conversion also showed a noticeable increase compared with the value “before switch”. In slow metabolizers, eGFR values did not differ noticeably between “before switch” and all other time points (Figure 3B).

In the multivariate analysis, a noticeable change of the eGFR over time pooled over both metabolism groups was observed (*p* = 0.003, Table S1). In this analysis, there was a noticeable eGFR increase in fast metabolizers at all time points compared to before the conversion. No noticeable changes in eGFR values (ΔeGFR) in slow metabolizers compared to before the conversion were found. When comparing the changes in eGFR (ΔeGFR) between fast and slow metabolizers (combination of main and interaction effects of tacrolimus metabolism group and time points), the fast group diverged the most from slow metabolizers within the first 10 days after the switch (4.15 (95% CI 0.54–7.76), *p* = 0.025). A further slight increase in the difference between the groups was found between D10 and M1 (1.84 (95% CI −1.39–5.07, *p* = 0.259).

**Figure 4:**
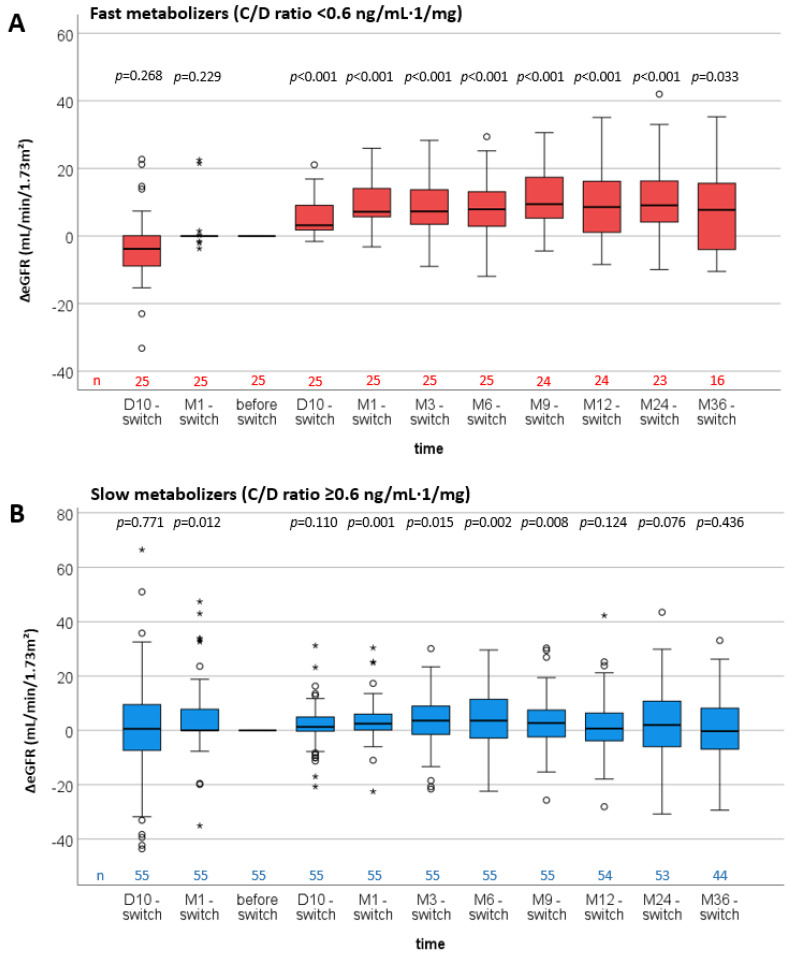
Boxplots of the differences in estimated glomerular filtration rates (ΔeGFR) of fast and slow Tac metabolizers (all time points—date of the switch). *p*-values are from Wilcoxon-signed rank tests for the dependent comparisons of eGFR values at each time point with the eGRF values at the time of the switch. The C/D ratio cut-off that characterized the metabolizer groups was changed to 0.6 ng/mL·1/mg. In this case, fast metabolizers developed an even more pronounced increase in ΔeGFR at D10 after conversion and all following time points compared with fast metabolizers defined by a C/D ratio cut-off of 1.05 ng/mL·1/mg (**A**). ΔeGFR of slow metabolizers also increased at M1–M9 after conversion to LCPT (**B**).

In a further examination, ΔeGFR values were compared between fast and slow metabolizers when a lower C/D ratio cut-off of 0.6 ng/mL·1/mg was chosen (<0.6 = fast metabolizers; ≥0.6 = slow metabolizers (Figure 4)). In this analysis, the increase in ΔeGFR was even more pronounced in fast metabolizers (Figure 4A). Interestingly, slow metabolizers were also found to have higher ΔeGFR at “M1 before switch” and “M1 to M9 after switch” (Figure 4B).

### 3.4. Complication Rates

No differences were observed in infection rates (CMV, BKV), BKVN, de novo development of post-transplant diabetes mellitus, CNIT, AR, or death between the groups at any time during the study (Table 4).

## 4. Discussion

In this study, we observed that the marked decline in renal function frequently observed in fast Tac metabolizers treated with IR- or ER-Tac after transplantation was prevented after switching to LCPT (Figure 3) [6,12].

Previously, we found that fast Tac metabolism in IR-treated RTx patients is associated with higher Tac peak levels and higher rejection and BKVN rates, leading to worse renal function compared with IR-treated slow metabolizers [5,6,11,13]. In our current observations, the renal function (eGFR) of the fast metabolizers was about 3 mL/min/1.73 m² lower than that of the comparison group before switching (Figure 2), but due to smaller cohorts in this study, the eGFR did not differ noticeably (*p* = 0.539) between groups. Others observed higher IF/TA levels in fast metabolizers than in slow metabolizers within one year of RTx [9,16]. Consistently, this was associated with worse graft survival and even mortality in fast metabolizers [6,8]. Although mortality was mainly caused by cardiovascular events, we did not observe differences in arterial stiffness or lipid levels between fast and slow metabolizers [11,17].

A major reason for switching from IR-Tac to LCPT was the variability in intra-patient trough levels (IPV, Table 2). IPV is associated with worse outcomes for several reasons [18]. LCPT has been shown to at least decrease Tac fluctuation and swing, and once-daily dosing is usually associated with increased adherence, both factors contributing to IPV, although study data on IPV in LCPT-treated patients are lacking [18,19].

LCPT was shown to be non-inferior to IR-Tac in RTx recipients and demonstrated lower efficacy failure rates in blacks [20,21]. Notably, blacks are often CYP3A5 expressers encoding tacrolimus-metabolizing cytochrome P450 3A5 enzymes and are therefore predominantly fast metabolizers [22,23]. Moreover, the fraction of fast metabolizers that are in the therapeutic Tac trough range is considerably higher for LCPT than for IR-Tac, potentially reducing the rejection rate in fast metabolizers (at least by trend, LCPT group: 23.2% vs. 36.6% IR-Tac group) [24]. In contrast, others showed that the rate of BKVN was lower in LCPT-treated recipients than in IR-Tac-treated ones [25]. This is interesting because we found an association between fast Tac metabolism and BKVN as well as BKV infection [5,13]. However, other studies found comparable BKV infection rates between LCPT and IR-Tac in de novo RTx recipients [21]. There were also no differences in metabolic parameters such as lipids or HbA1c between LCPT- and IR-treated patients [21,25]. Since the total exposure to the drug (area under the curve) and the trough level of fast and slow metabolizers and of LCPT and IR-Tac-treated patients are generally comparable, other factors—and it is probably not Tac metabolites that are similar in patients with low and high C/D ratios—must be responsible for the observed differences [11,19,22,26,27].

An important difference in the Tac-pharmacokinetics (PK) of patients with a low and a high C/D ratio is the peak concentration, which is thought to play a role in the development of Tac-associated nephrotoxicity [11]. In addition, it is noteworthy that the potentially harmful peak concentration is much lower with LCPT than with IR-Tac or ER-Tac [3]. In contrast to IR-Tac, LCPT treatment causes only one and not two Tac peaks and is not affected by chronopharmacokinetic effects [28]. An important point in this regard is that the major absorption site of LCPT is more distal in the gut than that of IR-Tac. As CYP3A5 expression decreases in the distal intestine, the bioavailability of LCPT is better than that of IR-Tac (as clearly shown by the reduced dosage after conversion, Table 3) and the dependence on CYP3A4/5 drug interference decreases significantly [29,30]. This is important because CYP3A5*1 is distinctly associated with fast Tac metabolism and CNIT [26]. Trofe-Clark et al. elegantly demonstrated in a study of stable RTx patients using a cross-over design that the PK of CYP3A5*1 expressers treated with LCPT, as opposed to IR-Tac treated individuals, was comparable to the PK of non-expressers. All treated patients benefited from the reduction in peak Tac concentration regardless of the CYP3A expression status, although the effect was significantly more pronounced in CYP3A5*1 expressers [22]. This may be one reason why fast metabolizers did not show pronounced eGFR loss during the 3-year follow-up period in the current study (Figure 2). Of note, fast metabolizers even showed a noticeable increase in renal function after the conversion to LCPT. These results are in line with a study on LTR that showed a noticeable recovery of eGFR 12 months after the conversion to LCPT (65.3 vs. 70.9 mL/min/1.73 m^2^; *p* < 0.001) [14].

As previously shown by us and others, eGFR was lower in fast IR- and ER-Tac metabolizers than in slow metabolizers already at M1 after RTx [6,7,8,12]. Since we did not perform PK analyses and assessed peak level, this is a limitation of our study. However, it should be remembered that the total absolute daily dose remained higher in fast metabolizers than in slow metabolizers, even under LCPT treatment; an effect that we also observed in our study (Table 3). Overall, it seems logical that the treatment with LCPT increases the C/D ratio when compared to IR-Tac calculated C/D ratios (Figure 3). This is in line with the literature [31,32].

Our study has several limitations. Because it is a single-center study with a limited number of patients, its power is limited. Further, due to the retrospective nature of our pilot study (first proof of concept), it can only be hypothesis-generating and encourage further prospective studies. Moreover, we did not determine the PK, peak, or IPV in our patients. Therefore, we can only make assumptions about the effects of LCPT on renal values. Fast metabolizers were mostly switched earlier to LCPT than slow metabolizers. This is not surprising, as the main reasons for switching were the side effects and IPV. Side effects and IPV are partly related to Tac dosage which are usually higher early after transplantation than later, due to different reasons, such as the intended trough level or decreasing steroid dosage [23]. However, steroid dosage was comparable in both groups (Table 3). Because we and others already saw the detrimental renal effects of Tac in the first months after RTx in fast metabolizers, this could be a reason for the more pronounced effect in the group with the low C/D ratio [5,10].

## 5. Conclusions

Our study revealed that switching from IR-Tac to LCPT increased the bioavailability of Tac, saved doses (and costs), and increased the C/D ratio. This may have mitigated the pronounced deterioration of renal function, particularly in fast metabolizers, which are more vulnerable in this respect. Because patients with a low C/D ratio are at risk for a worse outcome after RTx, we suggest calculating the C/D ratio early after RTx in patients treated with IR-Tac. Switching to LCPT was safe and could be beneficial early after RTx.

## Figures and Tables

**Table 1:** Patients’ characteristics.

	Fast Metabolizers *n* = 58	Slow Metabolizers *n* = 22	*p*-Value
age (years)	50.0 ± 16.1	49.8 ± 14.7	0.957 ^a^
sex (m/f), *n* (%)	37 (63.8%)/21 (36.2%)	13 (59.1%)/9 (40.9%)	0.797 ^b^
weight (kg)	80.8 ± 18.3	76.4 ± 15.1	0.274 ^a^
height (m)	1.77 ± 0.09	1.75 ± 0.09	0.267 ^a^
BMI (kg/m²)	25.5 ± 5.3	24.9 ± 4.3	0.570 ^a^
living donor transplantation	37 (63.8%)	16 (72.7%)	0.598 ^b^
ABO-i	13 (22.4%)	7 (31.8%)	0.399 ^b^
ESP transplantation	4 (6.9%)	1 (4.5%)	1 ^b^
DGF	6 (10.3%)	3 (13.6%)	0.700 ^b^
cold ischemic time (h)	7.4 ± 5.0	6.7 ± 4.3	0.539 ^a^
warm ischemic time (min)	35.9 ± 8.3	35.9 ± 9.2	0.968 ^a^
Number of Transplantations			
1	51 (87.9%)	19 (86.4%)	0.785 ^b^
2	6 (10.3%)	3 (13.6%)	
3	1 (1.7%)	0	
HLA MM			
0	13 (22.4%)	8 (36.4%)	0.462 ^b^
1–3	23 (39.7%)	7 (31.8%)	
4–6	22 (37.9%)	7 (31.8%)	
PRA > 20%	5 (8.6%)	2 (9.1%)	1 ^b^
CMV risk status			
low	13 (22.4%)	2 (9.1%)	0.105 ^b^
intermediate	34 (58.6%)	11(50.0%)	
high	11 (19.0%)	9 (40.9%)	
Donor Characteristics			
donor age (years)	52.7 ± 12.2	55.8 ± 9.7	0.246 ^a^
donor sex (m/f), n (%)	24 (41.4%)/34 (58.6%)	7 (31.8%)/15 (68.2%)	0.608 ^b^
Diagnosis of ESRD			
benign nephrosclerosis	4 (6.9%)	1 (4.5%)	0.345 ^b^
diabetic nephropathy	3 (5.2%)	1 (4.5%)	
glomerulonephritis	31 (53.4%)	14 (63.6%)	
chronic pyelonephritis	0	1 (4.5%)	
cystic nephropathy	14 (24.1%)	2 (9.1%)	
Alport syndrome	1 (1.7%)	0	
Mediterranean fever	0	1 (4.5%)	
congenital renal dysgenesis	4 (6.9%)	1 (4.5%)	
interstitial nephritis	1 (1.7%)	1 (4.5%)	
Comorbidities Before Transplantation			
arterial hypertension	56 (96.6%)	21 (95.5%)	1 ^b^
diabetes mellitus	8 (13.8%)	1 (4.5%)	0.432 ^b^

Data presented as mean ± standard deviation or absolute and relative frequencies. Abbreviations: BMI, body mass index; ABO-i, ABO incompatible transplantation; ESP, European Senior Program; DGF, delayed graft function; HLA MM, human leucocyte antigen mismatch; PRA, panel reactive antibodies; CMV, cytomegalovirus; ESRD, end-stage renal disease. Two-sided *p*-value from ^a^ Welch′s *t*-test or ^b^ Fisher′s exact test.

**Table 2:** Reasons for the switch from IR-Tac to LCPT.

	Fast Metabolizers *n* = 58	Slow Metabolizers *n* = 22	*p*-Value
infection	1 (1.7%)	1 (4.5%)	0.778
neurological disorder	2 (3.4%)	0	
acute rejection	1 (1.7%)	0	
CNIT	2 (3.4%)	2 (9.1%)	
DGF	2 (3.4%)	1 (4.5%)	
diabetes mellitus	1 (1.7%)	0	
trough level variation/avoidance of adverse effects	49 (84.5%)	18 (81.8%)	

IR-Tac, immediate-release tacrolimus; LCPT, LCP-tacrolimus; CNIT, calcineurin inhibitor toxicity; DGF, delayed graft function. The *p*-value is from the chi-squared test of independence.

**Table 3:** Immunosuppression.

	Fast Metabolizers *n* = 58	Slow Metabolizers *n* = 22	*p*-Value
**Prednisolone Dose after 1 Month**	17.5 (5–25)	15 (5–50)	0.680 ^a^
**Mycophenolate after 1 Month**			
mycophenolate mofetil, *n* (%)	30 (51.7%)	13 (59.1%)	0.621 ^b^
mycophenolate sodium, *n* (%)	28 (48.3%)	9 (40.9%)	
mycophenolate mofetil dose (mg)	1000 (500–2000)	1000 (500–2000)	0.932 ^a^
mycophenolate sodium dose (mg)	1440 (720–1440)	1080 (720–1440)	0.213 ^a^
**Tac Doses (mg)**			
IR-Tac M1	12 (5–20)	7 (4–12)	<0.001 ^a^
before switch (IR-Tac)	10.25 (3–18)	6.75 (1.5–17)	<0.001 ^a^
D10 LCPT	7 (1.5–14)	3.5 (1.8 11)	<0.001 ^a^
M1 LCPT	6 (1.5–13.5)	3 (1.5–11)	<0.001 ^a^
M3 LCPT	4.75 (1.5–12)	3 (1–8)	0.002 ^a^
M6 LCPT	4 (1.5–12)	2.5 (0.75–5)	0.001 ^a^
M9 LCPT	4 (1.5–11)	2.5 (1–6)	0.001 ^a^
M12 LCPT	3.63 (1.5–11)	2.25 (1–5)	0.001 ^a^
M24 LCPT	3.38 (1–9)	2.13 (0.75–5.5)	0.008 ^a^
M36 LCPT	3 (1–8.5)	2 (0.75–3.5)	0.016 ^a^
**Tac trough Levels (ng/mL)**			
IR-Tac M1	6.8 (2.4–15.9)	8.7 (6.8–13.5)	<0.001 ^a^
before switch (IR-Tac)	6.3 (2.4–12.3)	7.5 (3.9–13.5)	0.065 ^a^
D10 LCPT	7.2 (1.6–14.7)	6.3 (4.1–9.9)	0.026 ^a^
M1 LCPT	7.6 (1.5–19.5)	6.2 (3.7–12.9)	0.041 ^a^
M3 LCPT	7.3 (3.8–18.1)	6.7 (4.9–9.9)	0.311 ^a^
M6 LCPT	7.0 (2.7–11.4)	6.2 (4.0–10.4)	0.043 ^a^
M9 LCPT	6.5 (3.4–10.7)	6.3 (4.2–9.2)	0.992 ^a^
M12 LCPTT	6.2 (3.5–10.3)	6.05 (3.8–8.3)	0.224 ^a^
M24 LCPT	6.2 (4.0–10.7)	6.2 (2.1–9.4)	0.254 ^a^
M36 LCPT	5.5 (4.1–8.9)	5.40 (4.0–8.7)	0.698 ^a^
**Tac C/D Ratio (ng/mL·1/mg)**			
IR-Tac M1	0.64 (0.24–1.01)	1.25 (1.08–3.38)	<0.001 ^a^
before switch (IR-Tac)	0.66 (0.24–2.10)	1.15 (0.32–3.60)	0.001 ^a^
D10 LCPT	1.08 (0.33–4.90)	1.91 (0.40–4.06)	0.002 ^a^
M1 LCPT	1.24 (0.21–6.93)	2.23 (0.55–3.47)	0.010 ^a^
M3 LCPT	1.52 (0.55–4.93)	2.33 (0.94–6.60)	0.004 ^a^
M6 LCPT	1.58 (0.39–5.93)	2.65 (1.06–7.07)	0.007 ^a^
M9 LCPT	1.63 (0.40–5.07)	3.23 (1.23–6.30)	<0.001 ^a^
M12 LCPTT	1.74 (0.42–5.43)	2.75 (1.08–5.90)	0.007 ^a^
M24 LCPT	1.81 (0.64–5.40)	2.58 (0.96–6.27)	0.083 ^a^
M36 LCPT	1.85 (0.69–5.80)	2.65 (1.32–5.73)	0.026 ^a^

Data presented as median (25% quantile-75% quantile) or absolute and relative frequencies. Abbreviations: Tac, tacrolimus; C/D, concentration-to-dose; two-sided *p*-values from ^a^ Mann–Whitney U test or ^b^ Fisher´s exact test.

**Table 4:** Complications before and after switch.

	Fast Metabolizers *n* = 58	Slow Metabolizers *n* = 22	*p*-Value
CMV infection			
before switch to LCPT	8 (13.8%)	2 (9.1%)	0.719
after switch to LCPT (3 y-follow up)	3 (5.2%)	2 (9.1%)	0.612
BKV infection			
before switch to LCPT	3 (5.2%)	3 (13.6%)	0.338
after switch to LCPT (3 y-follow up)	1 (1.7%)	0	1
BKV nephropathy			
before switch to LCPT	1 (1.7%)	2 (9.1%)	0.182
after switch to LCPT (3 y-follow up)	0	0	-
CNIT			
before switch to LCPT	3 (5.2%)	2 (9.1%)	0.612
after switch to LCPT (3 y-follow up)	1 (1.7%)	0	1
acute rejection			
before switch to LCPT	12 (20.7%)	9 (40.9%)	0.089
after switch to LCPT (3 y-follow up)	8 (13.8%)	2 (9.1%)	0.719
death within 3 years after switch	2 (3.4%)	0	1
diabetes mellitus			
before switch to LCPT	1 (1.7%)	1 (4.5%)	0.477
after switch to LCPT (3 y-follow up)	0	0	-

CMV, cytomegalovirus; BKV, BK-virus; CNIT, calcineurin-inhibitor nephrotoxicity; LCPT, LCP-tacrolimus. *p*-values are from the Fisher’s exact test.

## Supplementary Materials

The following supporting information can be downloaded at: https://www.mdpi.com/article/10.3390/jcm11051290/s1, Table S1: Immunosuppression; Table S2: Model-based estimates of eGFR.

## References

[1] Tholking G., Gerth H.U., Schuette-Nuetgen K., Reuter S. (2017). Influence of tacrolimus metabolism rate on renal function after solid organ transplantation. World J. Transplant..

[2] Ong S.C., Gaston R.S. (2021). Thirty years of tacrolimus in clinical practice. Transplantation.

[3] Tremblay S., Nigro V., Weinberg J., Woodle E.S., Alloway R.R. (2017). A steady-state head-to-head pharmacokinetic comparison of all FK-506 (Tacrolimus) formulations (ASTCOFF): An open-label, prospective, randomized, two-arm, three-period crossover study. Am. J. Transplant..

[4] Kuypers D.R., Peeters P.C., Sennesael J.J., Kianda M.N., Vrijens B., Kristanto P., Dobbels F., Vanrenterghem Y., Kanaan N., Team A.S. (2013). Improved adherence to tacrolimus once-daily formulation in renal recipients: A randomized controlled trial using electronic monitoring. Transplantation.

[5] Tholking G., Fortmann C., Koch R., Gerth H.U., Pabst D., Pavenstadt H., Kabar I., Husing A., Wolters H., Reuter S. (2014). The tacrolimus metabolism rate influences renal function after kidney transplantation. PLoS ONE.

[6] Schutte-Nutgen K., Tholking G., Steinke J., Pavenstadt H., Schmidt R., Suwelack B., Reuter S. (2019). Fast tac metabolizers at risk (-) it is time for a C/D ratio calculation. J. Clin. Med..

[7] Nowicka M., Gorska M., Nowicka Z., Edyko K., Edyko P., Wislicki S., Zawiasa-Bryszewska A., Strzelczyk J., Matych J., Kurnatowska I. (2019). Tacrolimus: Influence of the posttransplant concentration/dose ratio on kidney graft function in a two-year follow-up. Kidney Blood Press Res..

[8] Jouve T., Fonrose X., Noble J., Janbon B., Fiard G., Malvezzi P., Stanke-Labesque F., Rostaing L. (2019). The TOMATO study (TacrOlimus MetabolizAtion in kidney TransplantatiOn): Impact of the concentration-dose ratio on death-censored graft survival. Transplantation.

[9] Egeland E.J., Reisaeter A.V., Robertsen I., Midtvedt K., Strom E.H., Holdaas H., Hartmann A., Asberg A. (2019). High tacrolimus clearance—A risk factor for development of interstitial fibrosis and tubular atrophy in the transplanted kidney: A retrospective single-center cohort study. Transpl. Int..

[10] Egeland E.J., Robertsen I., Hermann M., Midtvedt K., Storset E., Gustavsen M.T., Reisaeter A.V., Klaasen R., Bergan S., Holdaas H. (2017). High tacrolimus clearance is a risk factor for acute rejection in the early phase after renal transplantation. Transplantation.

[11] Tholking G., Schutte-Nutgen K., Schmitz J., Rovas A., Dahmen M., Bautz J., Jehn U., Pavenstadt H., Heitplatz B., Van Marck V. (2019). A Low tacrolimus concentration/dose ratio increases the risk for the development of acute calcineurin inhibitor-induced nephrotoxicity. J. Clin. Med..

[12] Tholking G., Filensky B., Jehn U., Schutte-Nutgen K., Koch R., Kurschat C., Pavenstadt H., Suwelack B., Reuter S., Kuypers D. (2021). Increased renal function decline in fast metabolizers using extended-release tacrolimus after kidney transplantation. Sci. Rep..

[13] Tholking G., Schmidt C., Koch R., Schuette-Nuetgen K., Pabst D., Wolters H., Kabar I., Husing A., Pavenstadt H., Reuter S. (2016). Influence of tacrolimus metabolism rate on BKV infection after kidney transplantation. Sci. Rep..

[14] Von Einsiedel J., Tholking G., Wilms C., Vorona E., Bokemeyer A., Schmidt H.H., Kabar I., Husing-Kabar A. (2020). Conversion from standard-release tacrolimus to MeltDose((R)) Tacrolimus (LCPT) improves renal function after liver transplantation. J. Clin. Med..

[15] Levey A.S., Stevens L.A., Schmid C.H., Zhang Y.L., Castro A.F., Feldman H.I., Kusek J.W., Eggers P., Van Lente F., Greene T. (2009). A new equation to estimate glomerular filtration rate. Ann. Intern. Med..

[16] Chamoun B., Torres I.B., Gabaldon A., Sellares J., Perello M., Castella E., Guri X., Salcedo M., Toapanta N.G., Cidraque I. (2021). Progression of interstitial fibrosis and tubular atrophy in low immunological risk renal transplants monitored by sequential surveillance biopsies: The influence of TAC exposure and metabolism. J. Clin. Med..

[17] Tholking G., Schulte C., Jehn U., Schutte-Nutgen K., Pavenstadt H., Suwelack B., Reuter S. (2021). The tacrolimus metabolism rate and dyslipidemia after kidney transplantation. J. Clin. Med..

[18] Kuypers D.R.J. (2020). Intrapatient variability of tacrolimus exposure in solid organ transplantation: A novel marker for clinical outcome. Clin. Pharmacol. Ther..

[19] Gaber A.O., Alloway R.R., Bodziak K., Kaplan B., Bunnapradist S. (2013). Conversion from twice-daily tacrolimus capsules to once-daily extended-release tacrolimus (LCPT): A phase 2 trial of stable renal transplant recipients. Transplantation.

[20] Bunnapradist S., Rostaing L., Alloway R.R., West-Thielke P., Denny J., Mulgaonkar S., Budde K. (2016). LCPT once-daily extended-release tacrolimus tablets versus twice-daily capsules: A pooled analysis of two phase 3 trials in important de novo and stable kidney transplant recipient subgroups. Transpl. Int..

[21] Budde K., Bunnapradist S., Grinyo J.M., Ciechanowski K., Denny J.E., Silva H.T., Rostaing L., Envarsus Study Group (2014). Novel once-daily extended-release tacrolimus (LCPT) versus twice-daily tacrolimus in de novo kidney transplants: One-year results of Phase III, double-blind, randomized trial. Am. J. Transplant..

[22] Trofe-Clark J., Brennan D.C., West-Thielke P., Milone M.C., Lim M.A., Neubauer R., Nigro V., Bloom R.D. (2018). Results of ASERTAA, a Randomized Prospective Crossover Pharmacogenetic Study of Immediate-Release Versus Extended-Release Tacrolimus in African American Kidney Transplant Recipients. Am. J. Kidney Dis..

[23] Schutte-Nutgen K., Tholking G., Suwelack B., Reuter S. (2018). Tacrolimus—Pharmacokinetic considerations for clinicians. Curr. Drug Metab..

[24] Suwelack B., Bunnapradist S., Meier-Kriesche U., Stevens D.R., Procaccianti C., Morganti R., Budde K. (2020). Effect of concentration/dose ratio in de novo kidney transplant recipients receiving LCP-tacrolimus or immediate-release tacrolimus: Post hoc analysis of a phase 3 clinical trial. Ann. Transplant..

[25] Glander P., Waiser J., Kasbohm S., Friedersdorff F., Peters R., Rudolph B., Wu K., Budde K., Liefeldt L. (2018). Bioavailability and costs of once-daily and twice-daily tacrolimus formulations in de novo kidney transplantation. Clin. Transplant..

[26] Kuypers D.R., Naesens M., de Jonge H., Lerut E., Verbeke K., Vanrenterghem Y. (2010). Tacrolimus dose requirements and CYP3A5 genotype and the development of calcineurin inhibitor-associated nephrotoxicity in renal allograft recipients. Ther. Drug Monit..

[27] Hryniewiecka E., Zegarska J., Zochowska D., Samborowska E., Jazwiec R., Kosieradzki M., Nazarewski S., Dadlez M., Paczek L. (2019). Dose-adjusted and dose/kg-adjusted concentrations of mycophenolic acid precursors reflect metabolic ratios of their metabolites in contrast with tacrolimus and cyclosporine. Biosci. Rep..

[28] Alloway R.R., Trofe-Clark J., Brennan D.C., Kerr J., Cohen E.A., Meier-Kriesche U., Stevens D.R., Moten M.A., Momper J.D. (2020). Chronopharmacokinetics and food effects of single-dose LCP-tacrolimus in healthy volunteers. Ther. Drug Monit..

[29] Tremblay S., Alloway R.R. (2017). Clinical evaluation of modified release and immediate release tacrolimus formulations. AAPS J..

[30] Huppertz A., Ott C., Bruckner T., Foerster K.I., Burhenne J., Weiss J., Zorn M., Haefeli W.E., Czock D. (2019). Prolonged-release tacrolimus is less susceptible to interaction with the strong CYP3A inhibitor voriconazole in healthy volunteers. Clin. Pharmacol. Ther..

[31] Rostaing L., Bunnapradist S., Grinyo J.M., Ciechanowski K., Denny J.E., Silva H.T., Budde K., Envarsus Study Group (2016). Novel once-daily extended-release tacrolimus versus twice-daily tacrolimus in de novo kidney transplant recipients: Two-year results of phase 3, double-blind, randomized trial. Am. J. Kidney Dis..

[32] Faravardeh A., Akkina S., Villicana R., Guerra G., Moten M.A., Meier-Kriesche U., Stevens D.R., Patel S.J., Bunnapradist S. (2021). Efficacy and safety of once-daily lcp-tacrolimus versus twice-daily immediate-release tacrolimus in adult hispanic stable kidney transplant recipients: Sub-Group analysis from a phase 3 trial. Ann. Transplant..

