# Supplementary material for: Improved Kidney Allograft Function after Early Conversion of Fast IR-Tac Metabolizers to LCP-Tac
Source: J Clin Med. 2022 Feb 26;11(5):1290. doi: 10.3390/jcm11051290 (PMC8911319; doi:10.3390/jcm11051290)
Supplement: Supplementary file 1 [file jcm-11-01290-s001.zip › jcm-1588207-supplementary.pdf]

Improved Kidney Allograft Function after Early Conversion of Fast IR-Tac Metabolizers to LCP-Tac

Gerold Thölking [1,2,*], Filiz Tosun-Koç [1], Ulrich Jehn [2], Raphael Koch [3], Hermann Pavenstädt [2], Barbara Suwelack [2], Stefan Reuter [2]

Table S1. Immunosuppression.

	**fast metabolizers n=58**	**slow metabolizers n=22**	**p-value**
**prednisolone dose after 1 month**	17.5 (5 - 25)	15 (5 - 50)	0.680[a]
**mycophenolate after 1 month**			
mycophenolate mofetil, n (%)	30 (51.7%)	13 (59.1%)	0.621[b]
mycophenolate sodium, n (%)	28 (48.3%)	9 (40.9%)	
mycophenolate mofetil dose (mg)	1000 (500 - 2000)	1000 (500 - 2000)	0.932[a]
mycophenolate sodium dose (mg)	1440 (720 - 1440)	1080 (720 - 1440)	0.213[a]
**Tac doses (mg)**			
IR-Tac M1	12 (5 - 20)	7 (4 - 12)	<0.001[a]
before switch (IR-Tac)	10.25 (3 - 18)	6.75 (1.5 - 17)	<0.001[a]
D10 LCP-T	7 (1.5 - 14)	3.5 (1.8 11)	<0.001[a]
M1 LCP-T	6 (1.5 - 13.5)	3 (1.5 - 11)	<0.001[a]
M3 LCP-T	4.75 (1.5 - 12)	3 (1 - 8)	0.002[a]
M6 LCP-T	4 (1.5 - 12)	2.5 (0.75 - 5)	0.001[a]
M9 LCP-T	4 (1.5 - 11)	2.5 (1 - 6)	0.001[a]
M12 LCP-T	3.63 (1.5 - 11)	2.25 (1 - 5)	0.001[a]
M24 LCP-T	3.38 (1 - 9)	2.13 (0.75 - 5.5)	0.008[a]
M36 LCP-T	3 (1 - 8.5)	2 (0.75 - 3.5)	0.016[a]
**Tac trough levels (ng/mL)**			
IR-Tac M1	6.8 (2.4 - 15.9)	8.7 (6.8 - 13.5)	<0.001[a]
before switch (IR-Tac)	6.3 (2.4 - 12.3)	7.5 (3.9 - 13.5)	0.065[a]
D10 LCP-T	7.2 (1.6 - 14.7)	6.3 (4.1 - 9.9)	0.026[a]
M1 LCP-T	7.6 (1.5 - 19.5)	6.2 (3.7 - 12.9)	0.041[a]
M3 LCP-T	7.3 (3.8 - 18.1)	6.7 (4.9 - 9.9)	0.311[a]
M6 LCP-T	7.0 (2.7 - 11.4)	6.2 (4.0 - 10.4)	0.043[a]
M9 LCP-T	6.5 (3.4 - 10.7)	6.3 (4.2 - 9.2)	0.992[a]
M12 LCPT-T	6.2 (3.5 - 10.3)	6.05 (3.8 - 8.3)	0.224[a]
M24 LCP-T	6.2 (4.0 - 10.7)	6.2 (2.1 - 9.4)	0.254[a]
M36 LCP-T	5.5 (4.1 - 8.9)	5.40 (4.0 - 8.7)	0.698[a]
**Tac C/D ratio (ng/mL·1/mg)**			
IR-Tac M1	0.64 (0.24 - 1.01)	1.25 (1.08 - 3.38)	<0.001[a]
before switch (IR-Tac)	0.66 (0.24 - 2.10)	1.15 (0.32 - 3.60)	0.001[a]
D10 LCP-T	1.08 (0.33 - 4.90)	1.91 (0.40 - 4.06)	0.002[a]
M1 LCP-T	1.24 (0.21 - 6.93)	2.23 (0.55 - 3.47)	0.010[a]
M3 LCP-T	1.52 (0.55 - 4.93)	2.33 (0.94 - 6.60)	0.004[a]
M6 LCP-T	1.58 (0.39 - 5.93)	2.65 (1.06 - 7.07)	0.007[a]

M9 LCP-T	1.63 (0.40 - 5.07)	3.23 (1.23 - 6.30)	<0.001[a]
M12 LCPT-T	1.74 (0.42 - 5.43)	2.75 (1.08 - 5.90)	0.007[a]
M24 LCP-T	1.81 (0.64 - 5.40)	2.58 (0.96 - 6.27)	0.083[a]
M36 LCP-T	1.85 (0.69 - 5.80)	2.65 (1.32 - 5.73)	0.026[a]
Data presented as median (25% quantile-75% quantile), or absolute and relative frequencies. Abbreviations: Tac, tacrolimus; C/D, concentration to dose; two-sided p-values from [a] Mann-Whitney U test or [b] Fisher´s exact test.			

Table S2. Model-based estimates of eGFR.

		Estimate	Lower 95% confidence limit	Upper 95% confidence limit	p-value
Difference of metabolism group fast vs slow combined over all time points		3.55	-4.50	-11.60	0.382
Effect of time combined over both metabolism groups					0.003
Interaction term of metabolism groups*time points					0.146
Least square mean differences between fast and slow metabolizer by time points (combination of main and interaction effects of tacrolimus metabolism group and time points)					
Before switch	fast vs slow	-0.72	-9.20	7.76	0.866
at D10	fast vs slow	3.43	-6.02	12.88	0.473
at M1	fast vs slow	5.27	-3.38	13.92	0.229
at M3	fast vs slow	5.28	-2.71	13.27	0.192
at M6	fast vs slow	4.82	-3.24	12.87	0.237
at M9	fast vs slow	3.98	-4.10	12.06	0.330
at M12	fast vs slow	3.91	-4.51	12.33	0.358
at M24	fast vs slow	2.51	-7.29	12.31	0.611
at M36	fast vs slow	3.51	-5.91	12.94	0.460
Least square means of the mean change between the time points (Δ) by metabolism group (combination of main and interaction effects of tacrolimus metabolism group and time points)					
fast metabolizers	Δ D10 vs before switch	4.23	2.11	6.34	<0.001
	Δ M1 vs before switch	6.98	4.84	9.11	<0.001
	Δ M3 vs before switch	6.69	4.32	9.05	<.0001
	Δ M6 vs before switch	7.62	5.05	10.19	<.0001
	Δ M9 vs before switch	7.38	4.82	9.94	<.0001
	Δ M12 vs before switch	6.05	3.24	8.87	<.0001
	Δ M24 vs before switch	6.52	3.17	9.88	<0.001
	Δ M36 vs before switch	6.60	2.67	10.55	0.001

slow metabolizers	Δ D10 vs before switch	0.08	-2.85	3.01	0.966
	Δ M1 vs before switch	0.97	-1.94	3.92	0.505
	Δ M3 vs before switch	0.69	-4.18	5.55	0.780
	Δ M6 vs before switch	2.08	-2.40	6.56	0.358
	Δ M9 vs before switch	2.68	-2.32	7.88	0.290
	Δ M12 vs before switch	1.42	-4.51	7.36	0.635
	Δ M24 vs before switch	3.29	-3.08	9.66	0.307
Least square mean differences in the change between time points (Δ) compared between metabolism groups (combination of main and interaction effects of tacrolimus metabolism group and time points)					
Δ D10 vs before switch	fast vs slow	4.15	0.54	7.76	0.025
Δ M1 vs D10	fast vs slow	1.84	-1.39	5.07	0.259
Δ M3 vs M1	fast vs slow	0.01	-3.95	3.97	0.996
Δ M6 vs M3	fast vs slow	-0.46	-3.76	2.84	0.781
Δ M9 vs M6	fast vs slow	-0.84	-3.49	1.81	0.531
Δ M12 vs M9	fast vs slow	-0.07	-2.72	2.58	0.959
Δ M24 vs M12	fast vs slow	1.40	-5.34	2.54	0.482
Δ M36 vs M24	fast vs slow	1.00	-2.72	4.73	0.593

Results of the linear mixed model. Selected parameter estimates and least square means for estimated glomerular filtration rate (eGFR) (mL/min/1.73m^2) are shown. Main effects of metabolism groups and time points and the interaction term were included as influencing factors. P-values are from Wald tests. Repeated measurements for each patient were modelled using SAS PROC MIXED by fitting a marginal linear mixed model with an unstructured variance-covariance matrix for the residuals with patient as subject and the order given by time. The empirical sandwich covariance estimator was applied. D10, day 10 after switch; M1-M36, month 1 – month 36 after switch.
